# Morphological, genomic and transcriptomic responses of *Klebsiella pneumoniae* to the last-line antibiotic colistin

**DOI:** 10.1038/s41598-018-28199-y

**Published:** 2018-06-29

**Authors:** Amy K. Cain, Christine J. Boinett, Lars Barquist, Janina Dordel, Maria Fookes, Matthew Mayho, Matthew J. Ellington, David Goulding, Derek Pickard, Ryan R. Wick, Kathryn E. Holt, Julian Parkhill, Nicholas R. Thomson

**Affiliations:** 1 Wellcome Trust Sanger Institute, Wellcome Genome Campus, Hinxton, Cambridge CB10 1SA UK; 2 Helmholtz Institute for RNA-based Infection Research, Würzburg, D-97080 Germany; 3 Department of Biology, Drexel University, Philadelphia, 19104 PA USA; 4 Public Health England, 61 Colindale Avenue, London, NW9 5EQ UK; 5 Centre for Systems Genomics, The University of Melbourne, Melbourne, VIC Australia; 6 Department of Biochemistry and Molecular Biology, Bio21 Molecular Science and Biotechnology Institute, The University of Melbourne, Melbourne, VIC Australia; 7 Department of Infectious and Tropical Diseases, London School of Hygiene and Tropical Medicine, London, United Kingdom; 8 Present Address: Chemistry and Biomolecular Sciences, Macquarie University, Sydney, Australia; 9 Present Address: Hospital for Tropical Diseases, Wellcome Trust Major Overseas Programme, Oxford University Clinical Research Unit, Ho Chi Minh City, Vietnam

**Keywords:** Bacterial genetics, Antimicrobial resistance

## Abstract

Colistin remains one of the few antibiotics effective against multi-drug resistant (MDR) hospital pathogens, such as *Klebsiella pneumoniae*. Yet resistance to this last-line drug is rapidly increasing. Characterized mechanisms of col^R^ in *K*. *pneumoniae* are largely due to chromosomal mutations in two-component regulators, although a plasmid-mediated col^R^ mechanism has recently been uncovered. However, the effects of intrinsic colistin resistance are yet to be characterized on a whole-genome level. Here, we used a genomics-based approach to understand the mechanisms of adaptive col^R^ acquisition in *K*. *pneumoniae*. In controlled directed-evolution experiments we observed two distinct paths to colistin resistance acquisition. Whole genome sequencing identified mutations in two colistin resistance genes: in the known col^R^ regulator *phoQ* which became fixed in the population and resulted in a single amino acid change, and unstable minority variants in the recently described two-component sensor *crrB*. Through RNAseq and microscopy, we reveal the broad range of effects that colistin exposure has on the cell. This study is the first to use genomics to identify a population of minority variants with mutations in a col^R^ gene in *K*. *pneumoniae*.

## Introduction

Antimicrobial resistance is an urgent threat to human health worldwide, and current treatment options are dwindling. Multi-drug resistant *Klebsiella pneumoniae* (MDR-Kp) was flagged by the World Health Organization and the Centre for Disease Control as “an urgent threat to human health” as it causes a range of high-mortality illnesses, including pneumonia, urinary tract infections and bloodstream infections, which are only treatable with a handful of last-line antibiotics, such as colistin. Colistin is a positively charged polypeptide antibiotic of the polymyxin class that binds to lipid A on the negatively charged bacterial cell surface, lowering the net charge, permeabilising the membrane and culminating in cell lysis^1^. Colistin was discovered in 1947 and first used as an antimicrobial in the late 1950s for the treatment of Gram-negative infections, but its use diminished in the 1970s due to nephrotoxicity and was replaced largely by aminoglycosides^2–4^. Recently, colistin was reintroduced into the clinical setting because it remains largely effective against MDR-Kp, as well as other Gram-negative bacilli including *Acinetobacter baumannii* and *Pseudomonas aeruginosa*, particularly as a component of combination therapy^3,5^. Worldwide colistin resistance (col^R^) rates are around 1.5% for *K*. *pneumoniae*, however, in some high-use countries this can reach up to 40% for MDR-Kp^6^.

In 2015, the first non-chromosomal col^R^ gene, *mcr-1*, was identified on plasmids from food-associated *Escherichia coli* throughout China and now is also found in Europe^7,8^. Mobile genetic elements (MGEs) have also been associated with patient-to-patient spread of col^R^ in hospitals^9,10^. Increased capsule production plays a role in providing antibiotic resistance in *K*. *pneumoniae* by acting as a physical barrier^11,12^. However, the major mechanisms of col^R^ in *K*. *pneumoniae* are due to intrinsic mutations, which can be selected by exposure to colistin. They usually involve SNPs in the two-component system (TCS) genes *phoPQ* or *pmrAB*, where the sensor component (PhoQ/PmrB) activates the regulator component (PhoP/PmrA) via phosphorylation in response to environmental signals^13^. In *K*. *pneumoniae*, the regulator component of both systems control expression of the *arnBCADTEF* (or *pmr*) operon which decreases the net charge of lipid A by attaching additional sugars, specifically 4-amino-4-deoxy-L-arabinose (L-Ara4N), thus preventing the positively charged colistin from binding lipid A^14–16^. Similarly, the product of the PmrAB-controlled gene *pmrC* was shown to decorate lipid A with phosphoethanolamine to provide tolerance to colistin^17^. In addition, col^R^ is also conferred by inactivation of the small lipoprotein MgrB, which represses *phoPQ* expression^13,18–21^. Multiple mutations in the sensory component of the TCS *crrAB*^22^, were recently shown to confer high level colistin resistance in *Klebsiella pneumoniae*^23^. The *crrAB* system acts via a modulator named *crrC* that interacts with PmrAB to alter *arn* expression^24^, and recently it was proposed that *crrB* mutations also activate a putative efflux pump to remove antibiotics from the cell^25^.

Heteroresistance can be defined as subsets of an otherwise isogenic bacterial population that display a range of susceptibilities to an antibiotic. More recently, a review by El-Halfawy and Valvano^26^ described the use of the term ‘heteroresistance’ to denote genetic changes in subpopulations of bacteria in acquired resistance to antimicrobials. Heteroresistance has been recognized as a characteristic marker of rapid resistance gain, often resulting in therapeutic failure^27^. However, its full impact on bacterial antibiotic resistance is poorly understood, because of the difficulty detecting bacterial subpopulations using standard laboratory methods^26^. Colistin heteroresistance has been phenotypically shown to occur in *K*. *pneumoniae*^28^ and the genetic basis of heteroresistance has been the subject of several of studies which show mutations (SNPs or tandem gene duplications) in TCSs confer col^R^ in Gram-negative strains^21,22,29,30^.

Here, we explore the genetic basis of intrinsic colistin heteroresistance, which has not yet been investigated on a whole-genome level. Taking a holistic approach, due to the complex nature of colistin resistance and its significance in treatment of MDR-Kp infections, we used multiple genomic approaches to examine the molecular changes associated with spontaneous col^R^ gain and maintenance in populations of *K*. *pneumoniae*. We combined whole genome sequencing, RNA sequencing (RNAseq) and electron microscopy to uncover the effects of colistin stress on these spontaneously col^R^ populations to better understand the impact of increasing colistin tolerance on cells grown *in vitro*. We also examined the phylogenetic distribution of the *crrB* gene across *K*. *pneumoniae* to investigate whether it is horizontally acquired.

## Results and Discussion

### Directed evolution experiments reveal multiple phenotypic and genotypic paths to col^R^

**Figure 1:**
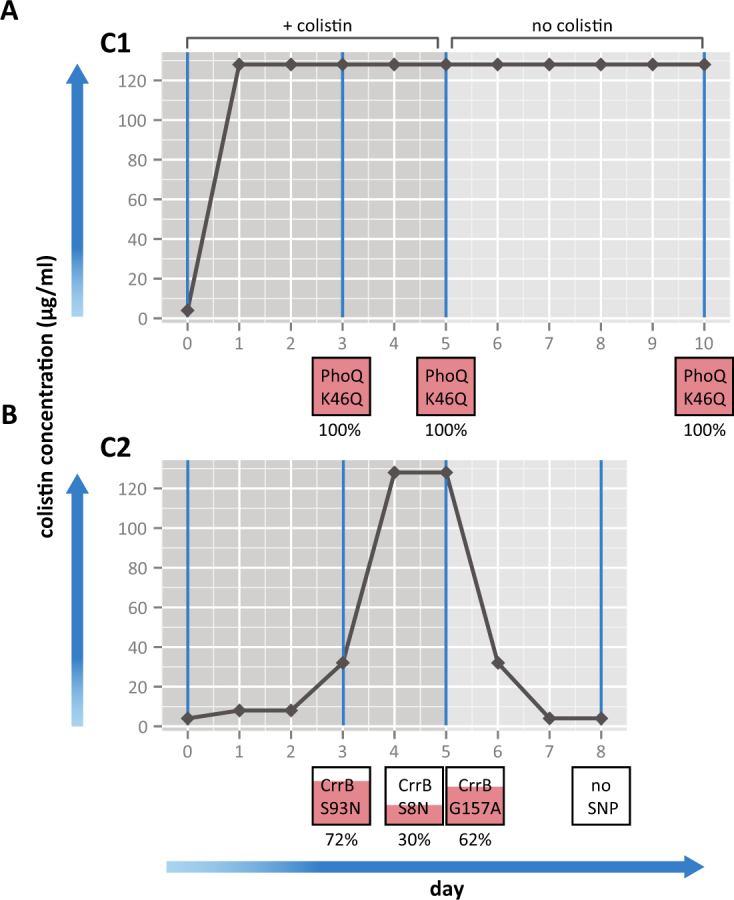
Bacterial growth with exposure to colistin sulfate and SNPs obtained at each day. The amount of bacterial growth on solid media was observed over a range of colistin concentrations (0–128 μg/ml) for 2 cultures of Ecl8 over 8–10 days for C1 (**A**) and C2 (**B**). The horizontal black lines represent bacterial growth on different concentrations of colistin over a 5 day time period, then growth after colistin selection is removed for another 5 days or until colistin sensitivity was reached. The boxes below represent the SNPs identified in each culture at D3, D5 or D10 and the amount of pink fill represents the proportion of the population harboring the SNP and this number is displayed below.

To induce spontaneous col^R^, two biological replicate cultures (denoted C1 and C2) of the colistin susceptible *K*. *pneumoniae* strain Ecl8^31^ were grown on solid media with increasing concentrations of colistin sulphate. Whole community growth (>20 colonies) was taken from the highest concentration of colistin for which there were colonies and re-plated on selective media containing a range of colistin concentrations (from 1–128 μg/ml in two-fold dilutions). This was repeated every day for five days and growth recorded (Table 1). We observed the emergence of two distinct patterns of colistin resistance (Fig. 1). Ecl8 Culture 1 (C1) became resistant to the highest colistin concentration tested (128 μg/ml) after one day of growth under colistin selection (Fig. 1A), whereas resistance for Culture 2 (C2) increased to the highest concentration (128 mg/L) over 4 days (Fig. 1B).

**Table 1:** Growth patterns observed on colistin plates for Ecl8 cultures C1 and C2.

	Culture	Day	Colistin concentration (µg/ml)^a^								SNPs detected compared to D0 (grown + col)					
			1	2	4	8	16	32	64	128	Gene	Position in gene	Ref	SNP	aa change	Proportion population (%)^b^
C1	+col	**D0**	+++	+++	**++**	−	−	−	−	−	−	−	−	−	−	−
		D1	+++	+++	+++	+++	+++	+++	+++	+						
		D2	+++	+++	+++	+++	+++	+++	+++	+++						
		**D3**	+++	+++	+++	+++	+++	+++	+++	**+++**	*phoQ* ^c^	136	T	C	K46Q	100
		D4	+++	+++	+++	+++	+++	+++	+++	+++						
		**D5**	+++	+++	+++	+++	+++	+++	+++	**+++**	*phoQ* ^c^	136	T	C	K46Q	100
C1	no col	D6	+++	+++	+++	+++	+++	+++	+++	+++						
		D7	+++	+++	+++	+++	+++	+++	+++	+++						
		D8	+++	+++	+++	+++	+++	+++	+++	+++						
		D9	+++	+++	+++	+++	+++	+++	+++	+++						
		**D10**	+++	+++	+++	+++	+++	+++	+++	**+++**	*phoQ* ^c^	136	T	C	K46Q	100
C2	+ col	**D0**	+++	+++	**++**	−	−	−	−	−	−	−	−	−	−	−
		D1	+++	+++	+++	+++	+	−	−	−						
		D2	+++	+++	+++	+	−	−	−	−						
		**D3**	+++	+++	+++	+++	+++	**+++**	−	−	*crrB* ^d,e^	278	G	A	S93N	71.8, 71.9
		D4	+++	+++	+++	+++	+++	+++	+++	+++						
		**D5**	+++	+++	+++	+++	+++	+++	+++	**+++**	*crrB* ^d^	470	G	A	G157A	61.5, 62.2
											*crrB* ^d^	23	G	C	S8N	23.8, 37.1
C2	no col	D6	+++	+++	+++	+++	+++	+++	−	−						
		D7	+++	+++	++	−	−	−	−	−						
		**D8**	+++	+++	**++**	−	−	−	−	−	none					

^a^Bacterial growth levels in 10 μl spot denoted by: +++ = lawn growth; ++ = 20–100 colonies; + = 4–20 colonies.

^b^Results for SNP analysis of DNA from the duplicate cultures are given.

^c^Also detected when grown without colistin selection.

^d^Not detected when grown without colistin selection.

^e^Annotated as “rstA regulator” or BN373_26561 (from positions 2777167–2778228 in Ecl8).

NB: The bold represent when the samples were extracted for sequencing.

### Mutations identified in phoQ and crrB by whole-genome sequencing

To understand the genotypic factors underlying col^R^ gain, we sequenced genomic DNA to identify SNPs in the Ecl8 C1 and C2 cultures at specific time points, day 3 (D3) and day 5 (D5). DNA was extracted from duplicate liquid cultures grown for a further day with and without colistin, and compared to the colistin naïve starting cultures at day 0 (D0) grown without selection (See Fig. 1 and Table 1). It was apparent that culture C1 possessed a SNP within *phoQ* (an A to G substitution at base position 136) at both time points (D3 and D5), with or without selection. This SNP represents a novel non-conservative amino acid change in PhoQ, with a positively charged lysine replacing a negatively charged glutamine at codon 46 (K46Q). This K46Q substitution is in close proximity to a mutation (T48I) previously shown to provide polymyxin resistance via loss of function of the PhoQ phosphatase domain, eliminating the ability of PhoQ to inactivate the PhoP activator^32,33^. We hypothesize that this K46Q substitution in PhoQ similarly disrupts the phosphatase domain, allowing continuous PhoP activation of downstream genes such as those that modify lipid A, thereby providing colistin resistance.

The consensus sequence derived from pooled cells of culture C2 from D3 and D5 was identical to that of the sequenced D0 colistin naïve cells for both of the duplicate extractions when using standard SNP calling methods based on mapping (present in >75% of reads, quality score >30; as in^34^). However, when alternative *de novo* assembly-based methods were used, three SNPs were identified in a subset of the sequence reads from the C2 cultures at D3 and D5 grown in the presence of colistin (see methods). When the mapping-based SNP detection cutoffs were relaxed (bases mismatching the reference and present in >5% of reads were considered), these three additional SNPs could be confirmed. However, these SNPs were not detected in the same cultures re-grown in the absence colistin selection by either variant calling method, nor were they present at any detectable level in the D0 starting cultures. All three SNPs detected in C2 fell within a single gene (locus ID BN373_26561; Table 1). The gene, BN373_26561, was 99% identical to *crrB*, previously shown to confer col^R^^22,23,25^.

**Figure 2:**
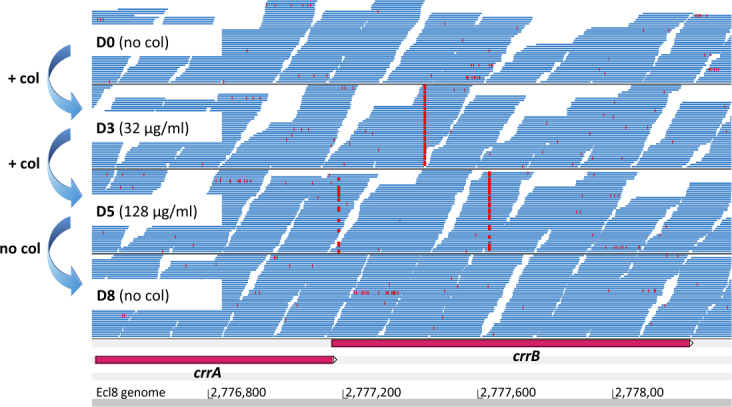
SNPs found in reads from heteroresistant C2 population in crrB. Screen shot of Artemis display with Bam files of the DNA sequencing reads, shown in blue, from D0 (colistin naïve), D3, D5 (colistin resistant) and D8 (colistin resistance lost) C2 cultures where bases differing from the reference (SNPs) are marked in red. The bp positions of the Ecl8 genome are shown below.

In C2 at D3, one SNP at position 278 bp within *crrB* gene (amino acid change S93N) was present in ~72% of the sequence reads (Fig. 2). Two other SNPs were detected in the C2 cells collected at D5. These were located at positions 23 and 470 bp of the *crrB* gene and were represented in 30% and 62% of reads and resulted in the non-synonymous changes G157A and S8N, respectively (Table 1; Fig. 2). Due to the short sequence read length, it was not possible to determine whether these 2 SNPs were present together in the same cell. None of these 3 SNPs in *crrB* have previously been associated with col^R^ and so we cannot be certain they are responsible for the col^R^ phenotype. However, this would be consistent with earlier findings that SNPs throughout the *crrB* gene can confer col^R^^24^.

Single colonies were isolated from the stored bulk C2 cultures from D3 and subjected to PCR and DNA sequencing of the *crrB* gene. Two colonies containing the SNP at 278 bp in *crrB* were identified and these were subjected to whole genome sequencing. Both colonies had a single non-synonymous SNP in *crrB* (S93N) compared to the Ecl8 wild-type and no additional mutations. These 2 colonies were tested for colistin susceptibly, both had an MIC of >128 μg/ml, which is 16x greater than the wild-type. Thus, we have confirmed that one of these single SNPs in *crrB* it is associated with a high level of colistin resistance in this *K*. *pneumoniae* strain. One isogenic mutant (“colony 9”) was used for further experimentation (see methods).

Unfortunately, after screening over 100 single colonies from stored frozen C2 cultures taken at D5, using PCR and DNA sequencing, we were unable to retrospectively recover any additional individual mutants: all possessed the wild type allele and not the *crrB* SNPs associated with resistance at D5 from the pooled cells. Thus, we could not perform additional experiments to prove that the minority populations of *crrB* mutants observed are causative of the colistin resistance phenotype observed. However, taken together with the evidence that mutations throughout *crrB* gene are proven to confer colistin resistance in *K*. *pneumoniae*^23^ and that a heteroresistant population of the *phoQ* mutation has also been shown to give a col^R^ phenotype in *K*. *pneumoniae*^29^, it is likely that this is the case.

### Stability of mutant populations associated with col^R^

To assay for the stability of the *phoQ* and *crrB* mutations in both the C1 and C2 col^R^ cultures, cells taken at D5 were re-passaged 5 times without antibiotic selection (Fig. 1). Susceptibility assays revealed C1 cultures to be phenotypically resistant (at 128 μg/ml) until D10. Whole genome sequencing (WGS) confirmed the presence of the mutation at bp 136 in *phoQ*, indicating that the *phoQ* mutation is stably maintained without selection.

Conversely, cells taken from C2 became fully susceptible to colistin after a single passage in the absence of selection at D6 (Fig. 1). None of the mutations seen previously in *crrB* were identified in the sequence of C2 cells cultures grown in the absence of selection when subjected to WGS. To determine whether the mutant was simply growing more slowly than wild type, a comparison of growth rates was performed using the method described in Hall *et al*.^35^. There was no significant difference (two-tailed student *t*-test, *P* < 0.1) in growth rate between the single *crrB* (S93N) mutant “colony 9” (3.38 × 10^−3^ OD/min, maxOD_600_: 0.665) and the wild-type Ecl8 (3.37 × 10^−3^ OD/min, maxOD_600_: 0.646). Thus, the reason that the *crrB* mutants were undetectable from culture after selection remains unclear and was not investigated further.

### Transcriptomic and morphological responses to colistin in heterogeneous populations

To further characterize the overall responses of the C2 heterogeneous populations, we grew the C2 cultures in ¼ MIC of colistin (D3; 16 mg/L, MIC: 64 mg/L and D5 64 mg/L, MIC; ≥128 mg/L) to mid-log phase to extract the RNA. Due to the remarkable out competition of the *crrB* gene SNP variant by wild-type genotypes we could not directly assay the overall transcriptional effect of the heterogeneous population without colistin stress. Therefore, we performed RNAseq of C2 at D3 and D5 exposed to colistin as well as the D0 colistin naïve starting culture without colistin. Growing the cells at ½ MIC (D3; 32 mg/L and D5; 128 mg/L) severely retarded growth of the cultures in broth, we assume due to the heterogenous nature of this resistance mechanism, therefore we opted to grow the cultures in ¼ MIC to obtain enough cell numbers for RNA extraction.

We compared the transcriptomic differences between the heterogeneous C3 and C5 populations and the colistin-naïve Ecl8 starting population (D0). Of the 4202 total Ecl8 genes assayed in C2, we detected 389 and 226 differentially expressed genes at D3 and D5, respectively, compared to D0. (Table S1, Dataset S1). Genes showing increased expression in the C2 heterogeneous populations at D3 and D5 included all the genes in the *arnA-T* operon involved in lipid-A modification. Other notable increases were four genes surrounding *crrB* (Fig. S1), including a response regulator (*crrA*), *crrC* (BN373_26541), a glycosyl transferase (BN373_26571) and an acridine efflux pump (BN373_26531) all had a log_2_FC of up to 9 (over 500-fold). Of the 27 known efflux systems in Ecl8, 4 showed increased expression under colistin selection: BN373_11321 (RND-family), BN373_15271 (MacA) and BN373_36071 (RND-family). These efflux systems all have been shown to act as multi-drug transporters^36^. Conversely, genes encoding the porins OmpA and OmpC, which allow passive antibiotic uptake^37^ showed decreased levels of expression in C2 at D3 and D5 (log_2_FC of −1.5 and −2 respectively). The European Nucleotide Accession (ENA) accessions for raw data for all genes and conditions can be found in Table S2.

**Figure 3:**
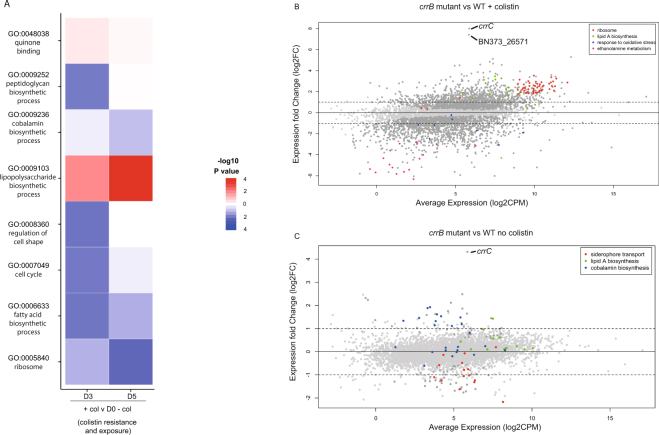
Overall effects of colistin exposure on colistin resistance Ecl8 cultures. (**A**) GO Terms derived from total differentially expressed genes in col^R^ heteroresistant poplution of C2. C2 D3, D5 grown with colistin selection compared to D0 colistin naïve cultures to give colistin exposure and resistance, and D3, D5 grown with colistin compared to the same day grown without colistin selection to give colistin stress effects. The represented GO terms had a GSEA FDR-corrected p-value less than 0.01 in any comparison. Heatmap colors represent −log10 p-values obtained from GSEA analysis of differentially expressed genes. Red colors indicate GO terms with higher expression relative to the comparator, blue colors lower expression. (**B**) Scatter plot of expression changes compared to average expression of single the SNP *crrB* mutant grown with and (**C**) without colistin exposed. Each black dot represents a gene, and genes belonging to relevant enriched pathways are marked in the colour given by the key. The fold change cut-offs used are marked with a black line.

Gene Ontology (GO) classification^38^ was used to summarize global changes in the transcriptome during colistin exposure. The most enriched GO term for increased expression in these cultures (summarised in Fig. 3A) was the “lipopolysaccharide biosynthetic process” (GO:0009103) and “quinone binding” (GO:0048038). The latter is consistent with a recently described secondary mechanism of action of polymyxins against Gram-negative bacteria which involves the inhibition of essential inner membrane respiratory enzymes such as NADH-quinone oxidoreductase^39^. Core metabolic functions showed a decreased expression in the “cobalamin biosynthetic process” (GO:0009236), relating to genes associated with vitamin B12 biosynthesis. Some unexpected functional groups were affected, spanning housekeeping, metabolic and morphological function, including: “regulation of cell shape” (GO:0008360), “cell cycle” (GO:0007049), “fatty acid biosynthetic process” (GO:0006633) and “ribosome” (GO:0005840), suggesting a broad downstream effect on the cell beyond specific colistin resistance mechanisms.

#### Morphological changes during colistin exposure

To further elucidate how the C2 colistin heterogeneous populations responded to colistin exposure, cells were viewed under transmission electron microscopy (TEM) to look for alterations in cell morphology (Fig. S2). Cells from the C2 heterogeneous population, taken from the plate with the highest concentration colistin at D5 were compared to colistin naïve cells from the D0 inoculation plate. C2 heterogeneous col^R^ populations when exposed to colistin revealed loss of membrane integrity and membrane blebbing. Other signs of stress were also apparent in these cells, including the presence of phage particles in the media and surrounding the bacterial cell membrane in addition to fimbriae. Also of note were the dark ion-dense granules in the colistin exposed C2 heterogeneous col^R^. These granules are known to accumulate at the end of active growth and are used for storage of excess sulphur, phosphate, carbohydrates and metal ions during phosphate or nitrogen starvation^40^. In addition the outline of extracellular polysaccharide (EPS) was observed more frequently in col^R^ cells.

Many of these TEM observations correlated with the RNA-seq data (see Fig. S2). This included increased expression of the Type 1 fimbriae biosynthetic cluster *fimACDFG* and genes from two of the three potential Ecl8 prophage (lambda and P2-like phages (BN373_1491-921 and BN373_33401-961 respectively;^41–43^), up to a log_2_FC of 7, or 128 fold increase (Dataset S1)^44^. Ion transport genes such as *mgtA* (BN373_02651) for magnesium transport or iron-chelate ABC transporters *fepAC*, and 4 genes of a FeCT family iron-chelate uptake transporter operon (BN373_10811-41) displayed increased expression, potentially related to the storage granules seen under TEM. As expected, OM proteins, involved in maintaining membrane integrity and stability during membrane stress, such as *slyB* and *yfgL* also showed increased expression, as well as general stress response genes, such as *dnaJK*. Overexpression of *slyB*, results in increased membrane permeability and the uptake of non-specific siderophores, a response related to stress response^45,46^. Although EPS production was observed in the TEM images, no transcriptomic changes were observed in the locus responsible for K2-like capsule formation (BN373_30991 to 31181).

### RNAseq on the crrB SNP mutant illustrates the col^R^ mechanism

To determine the specific effects of the *crrB* col^R^ SNP, we compared the transcriptome of the single *crrB* mutant, “colony 9” (*crrB*::S93N) to the wild type Ecl8, both grown in the presence and absence of selection (Fig. 3B,C). The only gene to have highly increased expression (>256 fold) in the *crrB*::S93N mutant without colistin exposure was *crrC* (Fig. 3C). This supports the col^R^ mechanism proposed by Cheng and colleagues^24^, where *crrB* activates the adjacent gene, *crrC*, and subsequently downstream lipidA modification genes via the *pmrAB* pathway. We also observed an increase in expression of between 2–4 fold of the *arn* lipid A modification genes as well as the other genes proximal to *crrB* (BN373_26531 and BN373_26571) predicted to encode an acridine efflux pump and glycosyltransferase, showing that these genes are activated in the *crrB*::S93N mutant. Non-synonymous mutations in *crrB* have previously described the increase in expression of lipid A modification genes, *crrC* and a putative glycosyltransferase, which were attributed to contribute to col^R^ ^22,24^.

Using GO terms to summarise these data: Lipid A biosynthesis genes showed increased expression (boxed in Fig. 3B) and conversely, a decreased expression in genes involved in siderophore transport and cobalamin biosynthesis. The specific reasons why these pathways were affected remains unknown, although recent work has shown that acquisition of col^R^ via *pmrB* mutations in *A*. *baumannii* results in decreased growth in iron limiting conditions^47^ and also that cobalamin synthesis and siderophore pathways interact as they compete for TonB-mediated transport across the outer membrane (OM)^48^.

When the *crrB*::S93N mutant was exposed to colistin (at 128 μg/ml) and compared to Ecl8 (grown in the absence of selection), *crrC* and a neighboring gene (BN373_26571) predicted to encode a glycostransferase, had increased expression (~256 fold). Also 9/16 genes in the *e*thanolamine *ut*ilization (*eut*) operon, encoding the ethanolamine catabolic pathway, showed decreased expression. This potentially makes more phosphoethanolamine available to be added to Lipid A by PmrC to change the cell charge^49^ associated with polymyxin resistance. Consistent with this *pmrC* also showed an increase in expression (9.8 fold).

Consistent with data from the heterogeneous culture, RNAseq of the *crrB*::S93N mutant showed an overall increase in Lipid A biosynthesis genes, as well as more pleiotropic effects including oxidative stress, changes in ribosomal genes as well as on core cellular processes.

### Phylogenetic distribution of crrB within Klebsiella pneumoniae

Previously *crrB* was found in only in a subset of *K*. *pneumoniae*^24^ contrasting with other TCS sensors that mediate col^R^, like *phoQ*, present in all *K*. *pneumoniae*. Moreover, it was hypothesized that *crrB* was acquired by *K*. *pneumoniae* via lateral gene transfer, due to its relatively low GC content^22^. To understand the significance of the *crrB* gene at the population level, we screened a global collection of representative *Klebsiella* isolates from a range of niches and sample types, including *K*. *pneumoniae* and the closely related species *K*. *quasipneumoniae* and *K*. *variicola* (dataset described in^50^).

**Figure 4:**
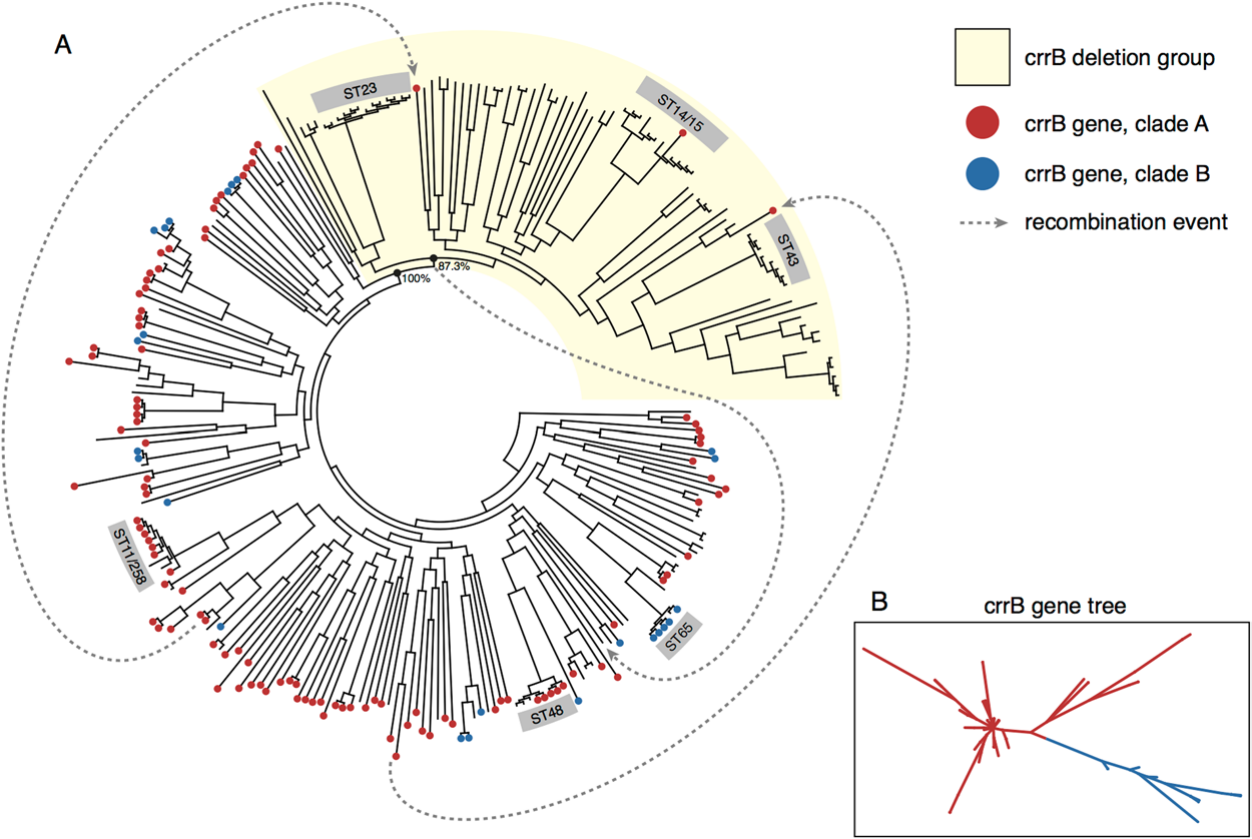
Phylogenetic tree of a KpI from a Global Klebsiella collection. (**A**) The dots on the ends of the branches represent whether the *crrB* gene is present in each isolate, red denotes lineages A and blue denotes lineage B. The boxed numbers on the outer ring shows the sequence type. The clade with KpI that has lost the *crrB* gene is shaded in beige. The grey lines represent an identified transfer of *crrB* and the arrow shows the inferred direction. (**B**) The 2 separate lineages of the *crrB* gene, based on SNP differences within the gene, are marked with blue and red colouring.

The *crrB* gene was detected in 54% (151/280) of all genomes screened (Dataset S2) including community and hospital isolates. However, of these *crrB*-containing isolates, 94% (142/151) belonged to *K*. *pneumoniae sensu stricto* (KpI) the species most often associated with human infection (^50^; Dataset S2). The frequency *K*. *quasipneumoniae* and *K*. *variicola* in *crrB*-containing isolates was only 5% and 1%, respectively. The phylogenetic distribution of *crrB* in the *K*. *pneumoniae* is shown in Fig. 4A, and reveals that the vast majority of strains carried this gene, including hospital-associated sequence types ST258/11, ST65 and ST48. However, *crrB* is only sporadically present in a single clade, including clinically important STs (ST23, ST14/15 and ST43), due to what appears to be a common basal deletion (shaded in Fig. 4A), indicating that *crrB* is not universally present in important disease causing STs.

In Ecl8, the *crrAB* operon is located within an ~11 kb region with a skewed GC-content (44.1% compared to the average of 56.9%; Fig. S1). This region includes an insertion sequence element, IS101 as well as additional genes that appear transcriptionally coupled to *crrB*. Across the different lineages, it appears that this *crr* locus was exchanged as a single unit between lineages having been repeatedly lost and re-acquired. However, there are no mobility functions, apart from the transposase associated with IS101 and so it is likely that exchange occurs through homologous recombination between the conserved regions flanking this locus.

The phylogeny of the *crrB* gene itself shows there are two major clades of the *crrB gene* (Fig. 4B) which is incongruent with the *K*. *pneumoniae* phylogeny (Fig. 4A) suggesting that additionally *crrB* alone is broadly exchanged across distant *K*. *pneumoniae* lineages by recombination. In support of this, we identified likely donors for two distinct horizontal gene transfer events that may explain the re-introduction of the *crrB* gene into the ostensibly *crrB*-negative clade (Fig. 4A). Taken together, these data suggest that *crrB* is ancestral to *K*. *pneumoniae*, was subsequently lost by deletion in one clade and the subject of intra-species recombination. No evidence of lateral gene transfer via mobile genetic elements was observed.

## Conclusions

*In vitro* induction of col^R^ resistance in *K*. *pneumoniae* identified known and novel SNPs in two genes known to confer colistin resistance. At the genotypic level we identified a novel SNP in, *phoQ*, as well as mutations in the *crrB* gene. One SNP, in *phoQ*, was present in 100% of the population, and was stable after selection was removed. This contrasted with the heterogeneous cultures, which carried mutations in the *crrB* gene. The *crrB* mutants were present in a maximum of ~70% of the total population under selection, but importantly never reached fixation and were not maintaining in the population after selection was removed. We show that exposing the *crrB* heterogeneous cultures to increasingly higher concentrations of colistin resulted in a range of transcriptional changes in core cellular functions, which correlated with morphological changes in the cells themselves. These far-reaching effects may explain why we do not observe col^R^ populations harboring *crrB* SNPs reaching fixation, presumably being outcompeted by isolates with a wild type *crrB* genotype in the absence of colistin. Certainly in other systems, susceptible wild-type genotypes were shown to outcompete the col^R^ variants in a heterogeneous population, including *pmrAB* variants in *Acinetobacter* spp.^51–53^. However, in our study, we found no significant difference in growth between the isogenic *crrB* SNP col^R^ mutant compared to a wild-type *crrB* carrying isolate. Further work is required to determine why these mutants are not maintained in the population and appear to be out-competed by the wild-type without selection in culture. The genetic basis of heteroresistance has only recently been described in several publications which show that mutations (SNPs or gene duplication) in TCSs, in a subset of the population, result in a heteroresistance phenotype. In most cases these result from alterations in expression in the lipid A modification genes^29,30,54^, whilst in another study no genetic basis was found to explain heteroresistance^55^. However in the later case, Band *et al*.^55^ observed a loss of col^R^ upon removal of selection thereby concluding it to be due to an unstable mutation. We propose that the heteroresistance mechanisms observed here mediated by mutations in *crrB* in only a subset of the population, may offer a new mechanism of achieving resistance during colistin exposure, whereby the mutant subpopulation would be quickly replaced by the wild-type strains in the absence of selection resulting in a reversion to pre-treatment sensitive levels as was observed by Band *et al*.^55^. We however could not determine the exact mechanism of heteroresistance, the increase in expression of lipid-A modification genes in the *crrB* heterogenous populations may explain the reason for the apparent loss of genotype and subsequent phenotype in the absence of selection. In addition, we observed the production of an EPS structures in the TEM images but no genetic mechanism to explain this phenotype. Perhaps the EPS provides a physical barrier, however further work will have to be done to prove this.

Although the phenotype is unstable, the potential to gain complete col^R^ upon exposure to the drug have further clinical implications with respect to the rapid nature of col^R^ acquisition by this mechanism.

Phylogenetic analysis of the distribution of *crrB* indicated that it was acquired earlier due to its basal position in *K*. *pneumoniae sensu stricto*, the principle species associated with human disease, but was lacking from the ancestors of the closely related species *K*. *variicola* and *K*. *quasipneumoniae*. Moreover, *crrB* was lost from one *K*. *pneumoniae* clade that includes important sequence types associated with liver abscesses and neonatal sepsis (ST23) as well as hospital associated MDR sequence types (ST14, ST15). Unlike *phoQ*, whilst there may be an ancestral function of *crrB*, it is not currently part of the core *K*. *pneumoniae* genome.

Finally, this work complements the recent discovery that a colistin resistance gene (*mcr-1*) can be carried on mobile extrachromosomal plasmids^7,55^ and illustrates that homologous recombination is another important mechanism facilitating the transfer of col^R^ genes (such as *crrAB*). This finding is particularly worrying and should reinforce the need to preserve our current effective antibiotics and dedicate resources to developing new ones.

One major limitation of our study is that because we did not to perform single gene knockouts and complementation studies, we could not definitively prove that the *crrB* minority variants were causative of the colistin resistance phenotype observed. Further, the instability of the crrB mutants without selection limited our ability to test the dynamics of these mutants in a population. However, we are continuing work to fully investigate the effect of *crrB* mutations on heteroresistant populations.

## Methods

### Inducing colistin resistance

Minimum Inhibitory Concentrations (MIC) of duplicate cultures of a colistin-sensitive ampicillin-resistant non-mucoid *Klebsiella pneumoniae* strain, Ecl8 (^31^; GenBank accession number HF5364824) and also two single colonies of the 258 bp *crrB* SNP mutant was determined using the British Society for Antimicrobial Chemotherapy (BSAC) agar dilution method^56^. To induce colistin resistance, duplicate cultures of Ecl8 were passaged daily in increasing concentrations of colistin sulfate from 1–128 μg/ml in serial dilutions on iso-sensitest agar (Oxoid), until they reached clinical col^R^ (128 μg/ml) over 5 days (~150 generations). We used iso-sensitest media throughout, as it has found to be most suitable for detecting colistin heteroresistance^57^. Approximately 10^9^ cells of initial inoculum was resuspended in 100 μl 0.9% saline, and 3 μl of this was spotted onto the colistin-containing agar plates which were grown overnight at 37 °C, and 10 μl was used to inoculate iso-sensitest broth for initial nucleic acid extraction (D0). Growth was taken from the highest concentration colistin plate where at least 20 colonies had growth and the entire colony spot was resuspended in 100 μl 0.9% saline and respotted onto another set of plates. At nucleic acid extraction days (D3, D5), 10 μl of the saline resuspension was also used to inoculate iso-sensitest broth + 1/2 MIC colistin and RNA was extracted mid-log phase, grown overnight, and DNA extracted. We also included positive and negative growth controls, to ensure viability of the selection.

## Electron Microscopy

The morphology of C2 col^R^ cells, taken from the 128 μg/ml colistin D5 plate, were compared to colistin naïve cells, taken from the colistin naïve starting plate at D0, by negative staining and fine structure analysis of ultrathin sections using transmission electron microscopy (TEM), on a Spirit Biotwin 120-kV TEM (FEI) and imaging with a F415 charge-coupled-device (CCD) camera (Tietz) For negative staining, cells were mixed with water and applied to a freshly glow-discharged, Formvar/carbon-coated EM grid and an equal volume of 3% ammonium molybdate with 1% trehalose. For the ultrathin sectional stains, cells were fixed with ruthenium hexamine trichloride (RHT) for 1 hour at room temperature in 2.5% glutaraldehyde in 0.05 M sodium cacodylate buffer with 0.7% RHT and 50 mM L-lysine monohydrochloride, followed by 3 buffered rinses, then re-fixed in 1% osmium tetroxide, dehydrated in an ethanol series (staining with 2% uranyl acetate at the 20% stage) and embedded in Epon araldite resin (Sigma). 50 μm ultrathin sections were cut on a UCT ultramicrotome EM (Leica), contrasted with uranyl acetate and lead citrate and analysed.

### Whole Genome Sequencing and SNP-calling

Whole community growth spots were resuspended in 100 µl PBS. 10 µl of this was used to inoculate cultures of 10 ml of iso-sensitest with 1/4 MIC colistin. DNA was extracted using a Promega Wizard Extraction Kit (Promega) and sequenced on an Illumina HiSeq2000 sequencer with 150 cycle paired-end runs. FASTQ files were deposited in the ENA, for accession number see Table S2. Sequences of isolates from D3, D5, and D10 were compared against their parental isolate D0 for C1 and C2, respectively to identify genomic variants conferring colistin resistance. SNP and indel variants were detected as previously described^58^ via a *de novo* assembly using SGA v0.9.19^59^ with built-in variant calling as well as a mapping approach using SMALT v0.7.4 with subsequent SNP calling^34^. To compare results between both methods variants were mapped back against Ecl8. Resulting variants were checked manually for accuracy. Single colonies of C2 from D3 were plated onto colistin containing agar and use as template DNA a PCR for the *crrB* gene (Primers in Table S3). We sequenced PCR products from 20 colonies, and 2 colonies containing the *crrB* SNP were subjected to whole genome sequencing on a 150 bp paired-end MiSeq run (Accession numbers in Table S1).

### Phylogenetic characterisation of crrB within K. pneumoniae

A phylogenetic analysis of *crrB* was performed to identify lineages of the gene. Sequence reads of a set of 328 population-wide isolates from Holt *et al*.^60^ were assembled *de novo* using an in-house assembly pipeline utilising Velvet v1.0.12^61^ and Velvet optimizer for initial contig assembly, scaffolding of contigs using SSPACE v2.0^62^, filling of gaps using GapFiller v1.11^63^, and a final mapping of reads against the scaffolds using SMALT v0.75 (H. Ponstingl and Z. Ning, manuscript in preparation; http://www.sanger.ac.uk/resources/software/smalt/). The presence of *crrB* was determined using a BLASTn search with a 98% cutoff as well as an in-house script which uses SMALT v0.75 to map the sequence of *crrB* back to the contigs using a 90% nucleotide acid identity cutoff. To detect recombination events in the *crrB* gene region, genomes were *de novo* assembled using SPAdes^64^ and suspected recombinant sequences were queried in against all genome assemblies using BLASTn^65^. High identity alignments from phylogenetically distant isolates were used to identify candidate recombinant DNA donors. Candidate donor sequences were then aligned to the query sequence using NUCmer^66^, SNP density was established, and the results were analysed with the Artemis Comparison Tool^67^. Alignments containing regions of very low SNP density compared to that across the rest of the genome were taken as evidence of recombination. Loss of the *crrB* gene due to recombination was further confirmed using the SPAdes assembly graphs. Bandage^68^ was used to view the assembly graphs and locate insertion sequences within *crrB* genetic context. Losses of the *crrB* gene due to nonsense mutation were identified using SeaView^69^. Phylogenetic analysis of the *crrB* gene was performed using RAxML^70^ to identify lineages (Fig. 4B). The two major clades of *crrB* were then plotted against the *K*. *pneumoniae* core genome phylogenetic tree generated previously^50^ and annotated with the inferred recombination events (Fig. 4A).

### RNAseq

RNA was extracted at OD 0.5 +/− 0.05, from the same D3, D5 and D0 C2 cultures as used in DNA extraction for SNP calling. Duplicate independent cultures were grown for each condition and time point to give technical replicates. RNA was extracted using a modified chloroform-based method (as previously described^71^) and depleted for ribosomal RNA (Ribo-Zero^TM^ Magnetic kit, Epicentre) prior to non-stranded sequencing using the TruSeq Illumina protocol. Samples were subsequently run on an Illumina HiSeq2500 sequencer for which an average of 1.75 Gb of raw reads in 100 base read pairs was obtained for each sample. The resulting FASTQ files were mapped against the *Klebsiella pneumonia* Ecl8 genome^31^. QPCR was used to confirm the trajectory of the fold-changes of 11 genes observed by RNAseq with primers listed in Table S3, as previously described^72^.

RNA was extracted at mid-log growth, from the same cultures as used for DNA extraction for whole genome sequencing. Due to the remarkable out competition of the *crrB* gene SNP variant by wild-type genotypes we could not directly assay the overall transcriptional effect of the heterogeneous population without colistin stress. Therefore we performed RNAseq of C2 at D3 and D5 exposed to colistin as well as the D0 colistin naïve starting culture. Transcriptomic analysis was also performed on the single *crrB* SNP mutant to determine the col^R^ mechanism, this mutant, grown in colistin, was compared to the wild-type strain grown in sub-inhibitory (1/4 MIC) concentrations of colistin.

#### Differential expression analysis

Read counts per feature were aggregated and further analysis was conducted in R. Read counts were normalized using the TMM normalization implemented in the EdgeR package version 3.4.2^73^. The Voom transformation^74^ was applied to the normalized read counts and differential expression analysis was performed using the Limma package version 3.18.13^75^. Read counts from C2 col^R^ cultures (D3 and D5) were compared to those from colistin naïve cultures (D0) to determine changes in gene expression after SNP acquisition and during colistin stress. RNA from each culture grown with and without colistin selection was also compared. Differentially expressed genes with a log_2_ fold change (logFC) of >1 (increased expression) or a logFC < −1 (decreased expression) and a Q-value < 0.05 were considered significant for this analysis.

#### Functional analysis of differentially expressed genes

Slimmed gene ontology (GO) terms were obtained for *K*. *pneumoniae* str. Ecl8 using the QuickGO interface^76,77^. Redundant entries were removed and Gene Set Enrichment Analysis (GSEA) analysis was then performed^38^ using GSEA version 2.1.0, run in ranked-list mode with enrichment statistic weighted on the per-gene logFCs calculated by voom, and GO-terms with a FDR corrected p-value less than 0.01 in any comparison are displayed in Fig. 3A.

### Availability of data and material

All sequence data is available at the European Nucleotide Archive (ENA) (http://www.ebi.ac.uk/ena) under the study accession number ERP004913. Specific sample accession numbers are given in Table S2. Other data generated or analysed during this study are included in this published article and its supplementary information files.

## Electronic supplementary material

Full Supplementary materials

Dataset 1

Dataset 2

## References

[1] Velkov T (2014). Surface changes and polymyxin interactions with a
resistant strain of Klebsiella pneumoniae. Innate Immun..

[2] Nation RL (2014). Consistent Global Approach on Reporting of Colistin
Doses to Promote Safe and Effective Use. Clin Infect Dis..

[3] Falagas ME, Kasiakou SK (2005). Colistin: the revival of polymyxins for the management
of multidrug-resistant gram-negative bacterial infections. Clin. Infect. Dis..

[4] Li J (2006). Colistin: the re-emerging antibiotic for
multidrug-resistant Gram-negative bacterial infections. Lancet Infectious Diseases.

[5] Biswas S, Brunel J-M, Dubus J-C, Reynaud-Gaubert M, Rolain J-M (2012). Colistin: an update on the antibiotic of the 21st
century. Expert Review of Anti-infective Therapy.

[6] Capone, a. *et al*. High rate of colistin resistance among patients with carbapenem-resistant Klebsiella pneumoniae infection accounts for an excess of mortality. *Clin*. *Microbiol*. *Infect*. **19** (2013). 10.1111/1469-0691.12070 23137235

[7] Liu Y (2015). Articles Emergence of plasmid-mediated colistin
resistance mechanism MCR-1 in animals and human beings in China: a
microbiological and molecular biological study. Lancet Infect. Dis..

[8] Skov RL, Monnet DL (2016). Plasmid-mediated colistin resistance (mcr-1gene):
three months later, the story unfolds. Eurosurveillance.

[9] Marchaim D (2011). Outbreak of colistin-resistant, carbapenem-resistant
Klebsiella pneumoniae in Metropolitan Detroit, Michigan. Antimicrob. Agents Chemother..

[10] Arduino SM (2012). Transposons and integrons in colistin-resistant clones
of Klebsiella pneumoniae and Acinetobacter baumannii with epidemic or sporadic
behaviour. J. Med. Microbiol..

[11] Pan, Y. J. *et al*. Capsular types of Klebsiella pneumoniae revisited by wzc sequencing. *PLoS One* **8** (2013). 10.1371/journal.pone.0080670 PMC3857182 24349011

[12] Arakawa Y (1995). Genomic organization of the Klebsiella pneumoniae cps
region responsible for serotype K2 capsular polysaccharide synthesis in the
virulent strain chedid. J. Bacteriol..

[13] Olaitan AO, Morand S, Rolain J-M (2014). Mechanisms of polymyxin resistance: acquired and
intrinsic resistance in bacteria. Front. Microbiol..

[14] Helander IM (1996). Characterization of lipopolysaccharides of
polymyxin-resistant and polymyxin-sensitive Klebsiella pneumoniae
O3. Eur. J. Biochem..

[15] Gunn JS (1998). PmrA-PmrB-regulated genes necessary for
4-aminoarabinose lipid A modification and polymyxin resistance. Mol. Microbiol..

[16] Mitrophanov, A. Y., Jewett, M. W., Hadley, T. J. & Groisman, E. a. Evolution and dynamics of regulatory architectures controlling polymyxin B resistance in enteric bacteria. *PLoS Genet*. **4** (2008). 10.1371/journal.pgen.1000233 PMC2565834 18949034

[17] Kim SH, Jia W, Parreira VR, Bishop RE, Gyles CL (2006). Phosphoethanolamine substitution in the lipid A of
Escherichia coli O157: H7 and its association with PmrC. Microbiology.

[18] Cannatelli A (2013). *In vivo* emergence
of colistin resistance in Klebsiella pneumoniae producing KPC-type
carbapenemases mediated by insertional inactivation of the PhoQ/PhoP mgrB
regulator. Antimicrob. Agents Chemother..

[19] Poirel L (2014). The mgrB gene as a key target for acquired resistance
to colistin in Klebsiella pneumoniae. J. Antimicrob. Chemother..

[20] The, H. C. *et al*. A high-resolution genomic analysis of multidrug- resistant hospital outbreaks of Klebsiella pneumoniae. *EMBO Mol Med*. **7**, 227–239 (2015). 10.15252/emmm.201404767 PMC4364942 25712531

[21] Zowawi HM (2015). Stepwise evolution of pandrug-resistance in Klebsiella
pneumoniae. Sci. Rep..

[22] Wright MS (2015). Genomic and Transcriptomic Analyses of
Colistin-Resistant Clinical Isolates of Klebsiella pneumoniae Reveal Multiple
Pathways of Resistance. Antimicrob. Agents Chemother..

[23] Jayol, A., Nordmann, P. & Brink, A. High level resistance to colistin mediated by various mutations in the crrB gene among carbapenemase-producing Klebsiella pneumoniae. *Antimicrob*. *Agents Chemother* **61** (2017). 10.1128/AAC.01423-17 PMC5655078 28874377

[24] Cheng, Y.-H., Lin, T.-L., Lin, Y.-T., Wang, J.-T. Amino acid substitutions of CrrB responsible for resistance to colistin through CrrC in Klebsiella pneumoniae, 10.1128/AAC.00009-16 (2016). 10.1128/AAC.00009-16 PMC4879426 27067316

[25] Cheng, Y.-H., Lin, T.-L., Lin, Y.-T. & Wang, J.-T. A putative RND-type efflux pump, H239_3064, contributes to colistin resistance through CrrB in Klebsiella pneumoniae. *J*. *Antimicrob*. *Chemother*. dky054–dky054 (2018). 10.1093/jac/dky054 PMC5961088 29506266

[26] El-Halfawy OM, Valvano Ma (2015). Antimicrobial Heteroresistance: an Emerging Field in
Need of Clarity. Clin. Microbiol. Rev..

[27] Falagas ME, Makris GC, Dimopoulos G, Matthaiou DK (2008). Heteroresistance: A concern of increasing clinical
significance?. Clin. Microbiol. Infect..

[28] Poudyal A (2008). *In vitro*
pharmacodynamics of colistin against multidrug-resistant Klebsiella
pneumoniae. J. Antimicrob. Chemother..

[29] Jayol A, Nordmann P, Brink A, Poirel L (2015). Heteroresistance to colistin in Klebsiella pneumoniae
associated with alterations in the PhoPQ regulatory system. Antimicrob. Agents Chemother.

[30] Lee, J.-Y., Choi, M.-J., Choi, H. J. & Ko, K. S. Preservation of Acquired Colistin Resistance in Gram-Negative Bacteria, 10.1128/AAC.01574-15 10.1128/AAC.01574-15 PMC4704156 26459897

[31] Fookes M, Yu J, De Majumdar S, Thomson N, Schneiders T (2013). Genome Sequence of Klebsiella pneumoniae Ecl8, a
Reference Strain for Targeted Genetic Manipulation. Genome Announc..

[32] Sanowar S, Martel A, Herve LM (2003). Mutational Analysis of the Residue at Position 48 in
the Salmonella enterica Serovar Typhimurium PhoQ Sensor Kinase. J. Bacteriol..

[33] Gunn JS, Miller SI (1996). PhoP-PhoQ activates transcription of pmrAB, encoding a
two-component regulatory system involved in Salmonella typhimurium antimicrobial
peptide resistance. J. Bacteriol..

[34] Harris SR (2010). Evolution of MRSA During Hospital Transmission and
Intercontinental Spread. Science (80-.).

[35] Hall BG, Acar H, Nandipati A, Barlow M (2014). Growth Rates Made Easy. Mol. Biol. Evol..

[36] Srinivasan, V. B., Singh, B. B., Priyadarshi, N., Chauhan, N. K. & Rajamohan, G. Role of novel multidrug efflux pump involved in drug resistance in Klebsiella pneumoniae. *PLoS One* **9** (2014). 10.1371/journal.pone.0096288 PMC4019481 24823362

[37] Hernández-Allés S (1999). Porin expression in clinical isolates of Klebsiella
pneumoniae. Microbiology.

[38] Subramanian A (2005). Gene set enrichment analysis: a knowledge-based
approach for interpreting genome-wide expression profiles. Proc. Natl. Acad. Sci. USA.

[39] Deris, Z. Z. *et al*. ORIGINAL ARTICLE A secondary mode of action of polymyxins against Gram-negative bacteria involves the inhibition of NADH-quinone oxidoreductase activity. **67**, 147–151 (2014). 10.1038/ja.2013.111 PMC3943757 24169795

[40] Shively, J. M. Inclusion Bodies of Prokaryotes. *Annu*. *Rev*. *Microbiol*. 167–187 (1974). 10.1146/annurev.mi.28.100174.001123 4372937

[41] Moreno Switt, A. I. *et al*. Salmonella phages and prophages: genomics, taxonomy, and applied aspects. *Salmonella Methods and Protocols* **1225** (2015). 10.1007/978-1-4939-1625-2_15 25253259

[42] Kropinski AM, Sulakvelidze A, Konczy P, Poppe C (2007). Salmonella phages and prophages–genomics and practical
aspects. Methods Mol. Biol..

[43] Thomson N (2004). The role of prophage-like elements in the diversity of
Salmonella enterica serovars. J. Mol. Biol..

[44] Zhou Y, Liang Y, Lynch KH, Dennis JJ, Wishart DS (2011). PHAST: A Fast Phage Search Tool. Nucleic Acids Res..

[45] Plesa, M., Hernalsteens, J., Vandenbussche, G. & Ruysschaert, J. The SlyB outer membrane lipoprotein of Burkholderia multivorans contributes to membrane integrity **157**, 582–592 (2006). 10.1016/j.resmic.2005.11.015 16500084

[46] Baumler AJ, Hantke K (1992). A lipoprotein of Yersinia enterocolitica facilitates
ferrioxamine uptake in Escherichia coli. J. Bacteriol..

[47] López-Rojas, R., García-Quintanilla, M., Labrador-Herrera, G., Pachón, J. & McConnell, M. J. Impaired growth under iron-limiting conditions associated with the acquisition of colistin resistance in Acinetobacter baumannii. *Int*. *J*. *Antimicrob*. *Agents*, 10.1016/j.ijantimicag.2016.03.010 (2016). 10.1016/j.ijantimicag.2016.03.010 27179817

[48] Kadner RJ, H. KJ (1995). Mutual Inhibition of Cobalamin and Siderophore Uptake
Systems Suggests Their Competition for TonB Function. J. Bacteriol..

[49] Raetz CRH, Reynolds CM, Trent MS, Bishop RE (2007). Lipid A modification systems in gram-negative
bacteria. Annu. Rev. Biochem..

[50] Holt KE (2015). Genomic analysis of diversity, population structure,
virulence, and antimicrobial resistance in Klebsiella pneumoniae, an urgent
threat to public health. Proc. Natl. Acad. Sci. USA.

[51] Beceiro A (2014). Biological cost of different mechanisms of colistin
resistance and their impact on virulence in acinetobacter
baumannii. Antimicrob. Agents Chemother..

[52] Snitkin ES (2013). Genomic insights into the fate of colistin resistance
and Acinetobacter baumannii during patient treatment. Genome Res..

[53] Falagas ME, Rafailidis PI, Matthaiou DK (2010). Resistance to polymyxins: Mechanisms, frequency and
treatment options. Drug Resist. Updat..

[54] Hjort, K., Nicoloff, H. E. & Andersson, D. I. Unstable tandem gene amplification generates heteroresistance (variation in resistance within a population) to colistin in Salmonella enterica, 10.1111/mmi.13459. 10.1111/mmi.13459 27381382

[55] Band VI (2016). Antibiotic failure mediated by a resistant
subpopulation in Enterobacter cloacae. Nat. Microbiol..

[56] Andrews, J. M., Working, B. & Testing, S. JAC BSAC standardized disc susceptibility testing method. *J. Antimicrob. Chemother*. **48**, Suppl. S1, 43–57 (2001). 10.1093/jac/48.suppl_1.43 11420336

[57] Lo-Ten-Foe JR, De Smet AMGA, Diederen BMW, Kluytmans JAJW, Van Keulen PHJ (2007). Comparative evaluation of the VITEK 2, disk diffusion,
etest, broth microdilution, and agar dilution susceptibility testing methods for
colistin in clinical isolates, including heteroresistant Enterobacter cloacae
and Acinetobacter baumannii strains. Antimicrob. Agents Chemother..

[58] Dordel J (2014). Novel determinants of antibiotic resistance:
Identification of mutated Loci in Highly methicillin-resistant subpopulations of
methicillin-resistant Staphylococcus aureus. MBio.

[59] Simpson, J. T. *et al*. Efficient de novo assembly of large genomes using compressed data structures sequence data. 549–556, 10.1101/gr.126953.111 (2012). 10.1101/gr.126953.111 PMC3290790 22156294

[60] Holt, K. E. *et al*. Diversity, population structure, virulence and antimicrobial resistance of Klebsiella pneumoniae, an urgent threat to public health. *PNAS* (2015). 10.1073/pnas.1501049112 PMC4500264 26100894

[61] Zerbino DR, Birney E (2008). Velvet: Algorithms for de novo short read assembly
using de Bruijn graphs. Genome Res..

[62] Boetzer M, Henkel CV, Jansen HJ, Butler D, Pirovano W (2011). Scaffolding pre-assembled contigs using
SSPACE. Bioinformatics.

[63] Boetzer M, Pirovano W (2012). Toward almost closed genomes with
GapFiller. Genome Biol..

[64] Bankevich A (2012). SPAdes: A New Genome Assembly Algorithm and Its
Applications to Single-Cell Sequencing. J. Comput. Biol..

[65] Camacho C (2009). BLAST+: architecture and applications. BMC Bioinformatics.

[66] Kurtz S (2004). Versatile and open software for comparing large
genomes. Genome Biol..

[67] Carver TJ (2005). ACT: The Artemis comparison tool. Bioinformatics.

[68] Wick RR, Schultz MB, Zobel J, Holt KE (2015). Bandage: Interactive visualization of de novo genome
assemblies. Bioinformatics.

[69] Gouy M, Guindon S, Gascuel O (2010). SeaView version 4: A multiplatform graphical user
interface for sequence alignment and phylogenetic tree building. Mol. Biol. Evol..

[70] Stamatakis A (2014). RAxML version 8: A tool for phylogenetic analysis and
post-analysis of large phylogenies. Bioinformatics.

[71] Vergara-Irigaray M, Fookes MC, Thomson NR, Tang CM (2014). RNA-seq analysis of the influence of anaerobiosis and
FNR on Shigella flexneri. BMC Genomics.

[72] De Majumdar S (2015). Elucidation of the RamA Regulon in Klebsiella
pneumoniae Reveals a Role in LPS Regulation. PLOS Pathog..

[73] Robinson MD, McCarthy DJ, Smyth GK (2009). edgeR: A Bioconductor package for differential
expression analysis of digital gene expression data. Bioinformatics.

[74] Law CW, Chen Y, Shi W, Smyth GK (2014). Voom: precision weights unlock linear model analysis
tools for RNA-seq read counts. Genome Biol..

[75] Smyth, G. K. In *Bioinfo*rmati*cs* and *Computational Biology Solutions Using R and Bioconductor SE -* 23 (eds Gentleman, R., Carey, V., Huber, W., Irizarry, R. & Dudoit, S.) 397–420, 10.1007/0-387-29362-0_23 (Springer New York, 2005).

[76] Binns D (2009). The Gene Ontology - Providing a Functional Role in
Proteomic. Studies..

[77] Caspi R (2014). The MetaCyc database of metabolic pathways and enzymes
and the BioCyc collection of Pathway/Genome Databases. Nucleic Acids Res..

