# Supplementary material for: Morphological, genomic and transcriptomic responses of Klebsiella pneumoniae to the last-line antibiotic colistin
Source: Sci Rep. 2018 Jun 29;8:9868. doi: 10.1038/s41598-018-28199-y (PMC6026146; doi:10.1038/s41598-018-28199-y)
Supplement: Supplementary file 1 — Full Supplementary materials [file 41598_2018_28199_MOESM1_ESM.pdf]

Morphological, genomic and transcriptomic responses of *Klebsiella pneumoniae* to the last-line antibiotic colistin

Authors

Amy K. Cain, Christine J. Boinett, Lars Barquist, Janina Dordel, Maria Fookes, Matthew Mayho, Matthew J. Ellington, David Goulding, Derek Pickard, Ryan R. Wick, Kathryn E. Holt, Julian Parkhill & Nicholas R. Thomson

Supplementary Information

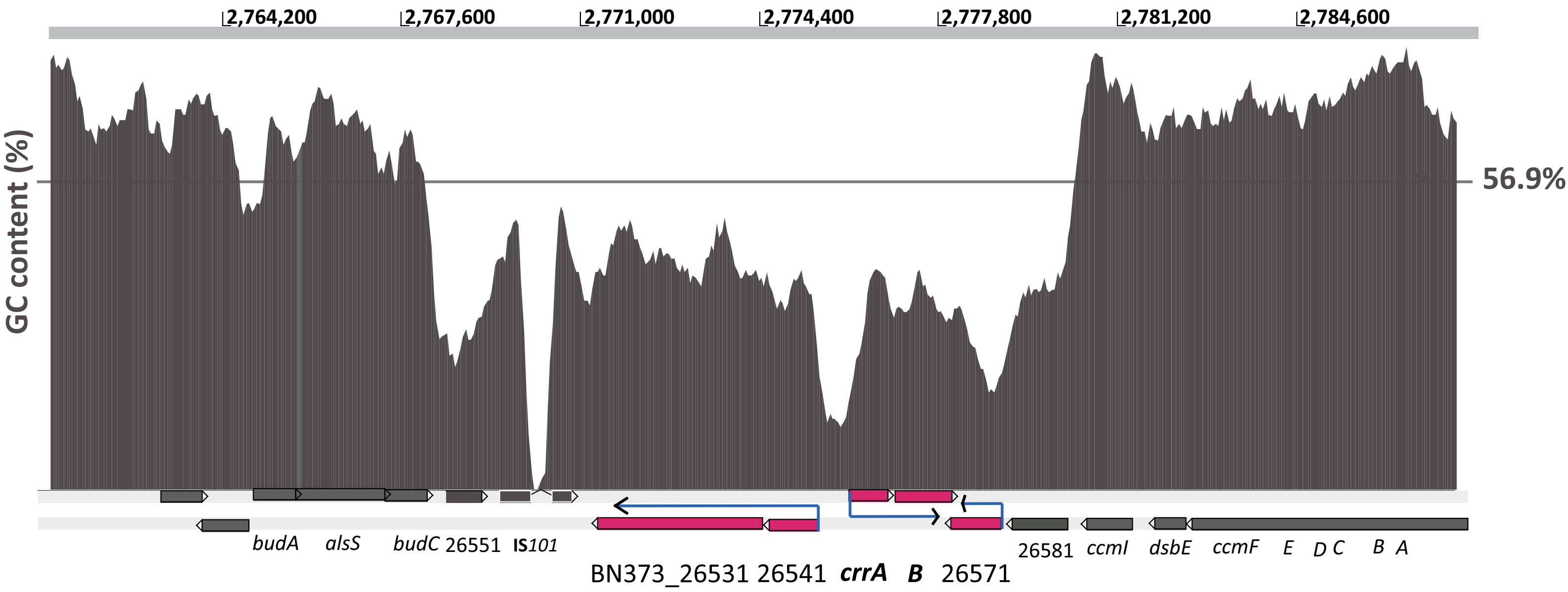

Figure S1. The crrB gene and its context within Ecl8. Artemis screen shot with the GC content of each gene shown above, with the horizontal line representing the average GC content of Ecl8. Genes surrounding crrB with increased expression during colistin exposure are marked in pink and the transcriptional units of these genes (as predicted using BioCyc: http://biocyc.org/) are shown by blue thin arrows.

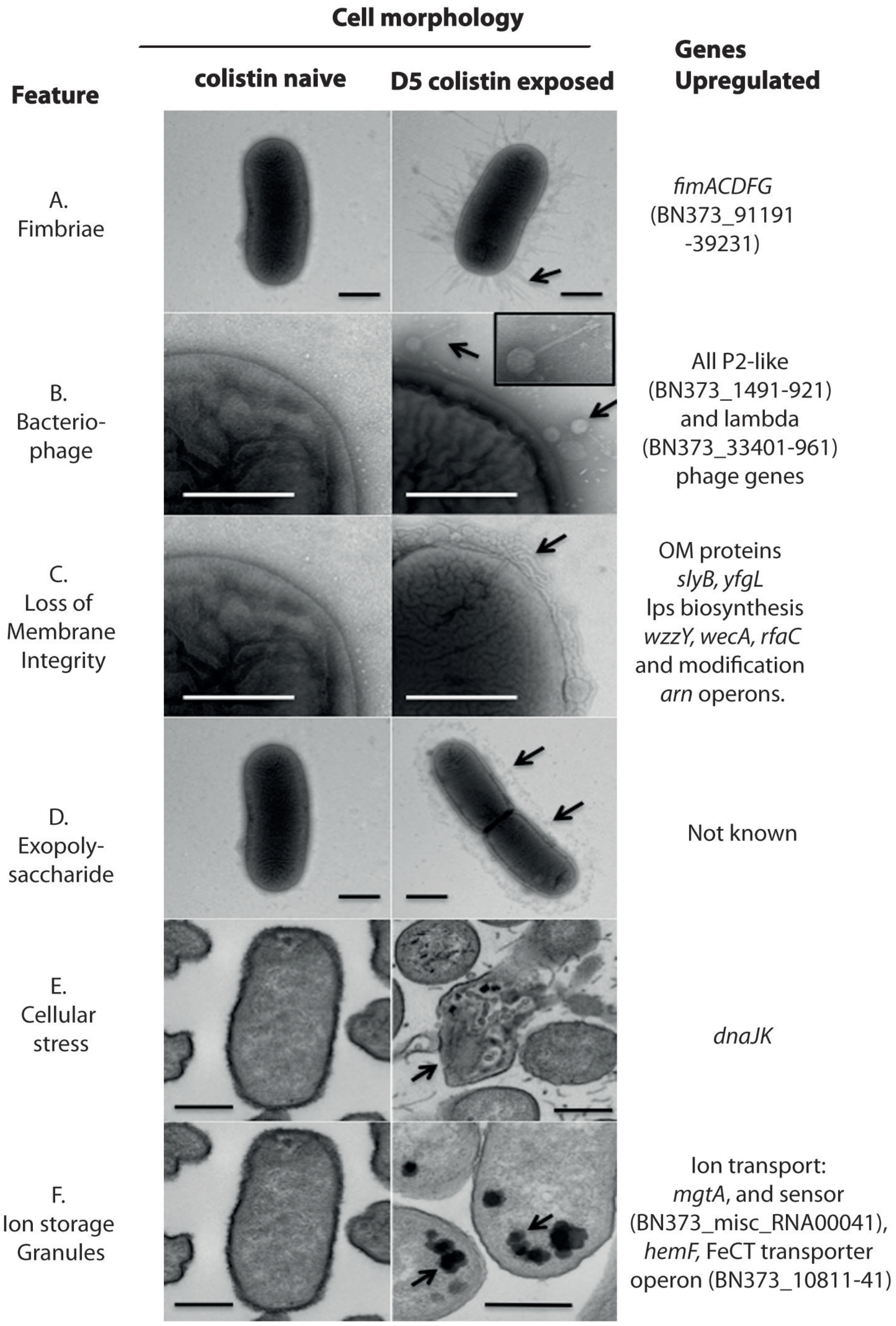

Figure S2. TEM and RNAseq comparison of colistin naïve cultures and colR cells under colistin exposure. We imaged 43 and 30 field of view using the capsule stain (A-D) and ultrathin sectioning (E-F) representative of total cells (from D0 control and D5 EM2 + col) and the above images our observations. For B, D, F these were representative of the majority of images observed, and for A, C, E, F these were only seen in the D5 heteroresistant cultures grown with colistin. For each morphological feature, any genes that showed differential expression of D5 + colistin compared to D0 naïve cultures are given on the right. White scale bar shown represents 0.5um and arrows indicate the difference between the colistin exposed and non-exposed cells highlighted for each feature.

Table S1. Changes in differential expression for known colistin resistance genes in log2 fold change (log_2FC) of raw read counts of bulk cultures for each day.

			Heteroresistant C2 D3 + 32mg/L col	Heteroresistant C2 D5 + 128mg/L col		C2 Mutant + 128mg/L col
**Col^R genes**	**Locus ID**	**Comparison**	**log_2FC**	**log_2FC**	**Comparison**	**log_2FC**
**Regulators**						
phoQ	BN373_17521	v no col	**3.30**	1.78	v WT no col	*0.11^*
		v D0	**2.68**	1.57	v WT + col	*0.22^*
phoP	BN373_17531	v no col	**3.52**	**2.02**	v WT no col	*0.15^*
		v D0	**2.62**	1.82	v WT + col	*-0.32*
mgrB	BN373_29261	v no col	**3.16**	1.37	v WT no col	*0.086^*
		v D0	**2.36**	1.27	v WT + col	*0.92^*
pmrB	BN373_13691	v no col	0.78	**5.03**	v WT no col	*0.59^*
		v D0	1.04	**4.69**	v WT + col	3.03
pmrA	BN373_13701	v no col	*0.45^*	**4.93**	v WT no col	0.55
		v D0	0.81	**4.91**	v WT + col	3.03
pmrC	BN373_13711	v no col	0.93	**5.65**	v WT no col	0.65
		v D0	0.72	**5.29**	v WT + col	3.27
pmrD	BN373_30261	v no col	**2.19**	**2.00**	v WT no col	*0.48^*
		v D0	**2.90**	**2.06**	v WT + col	0.58
crrB	BN373_26561	v no col	1.56	**2.64**	v WT no col	*0.71^*
		v D0	**3.17**	**3.52**	v WT + col	2.66
crrA	BN373_26551	v no col	*1.43^*	*1.25^*	v WT no col	*0.68^*
		v D0	**2.96**	**2.55**	v WT + col	2.58
**Lipid A modification**						
arnA-T (range)	arnA-T (range)	v no col	**6.47 to 4.54**	**2.69 to 2.54**	v WT no col	0.16 to 1.42
	BN373_44781-821	v D0	**5.60 to 4.53**	**6.53 to 5.17**	v WT + col	2.39 to 3.46
pagP	BN373_12351	v no col	**3.49**	1.65	v WT no col	0.56
		v D0	**2.96**	**2.55**	v WT + col	3.16
**Efflux**						
RND-family	BN373_11321	v no col	**2.46**	**2.02**	v WT no col	*0.002^*
		v D0	**3.76**	**2.42**	v WT + col	-1.21
macA	BN373_15271	v no col	**2.91**	1.60	v WT no col	-0.18
		v D0	**2.29**	1.62	v WT + col	0.11
oqxA	BN373_36071	v no col	*0.09^*	**2.32**	v WT no col	-0.35
		v D0	*1.11^*	**3.43**	v WT + col	-0.43
***crrB*-containing region**						
acridine pump	BN373_26531	v no col	*0.83^*	**6.09**	v WT no col	1.45
		v D0	*0.98^*	**6.37**	v WT + col	4.67
crrC	BN373_26541	v no col	*1.56^*	**8.67**	v WT no col	4.32
		v D0	**2.82**	**9.12**	v WT + col	8.02
glycosyltransferase	BN373_26571	v no col	**1.97**	**7.45**	v WT no col	1.65
		v D0	**3.95**	**8.09**	v WT + col	7.42

^ = not statistically significant (adjusted P-value >0.05)
Italic font denotes results not statistically significant (adjusted P-value of >0.05); bold denotes a significant expression change (log_2fold Change >2); blue denotes cells with both; light blue is changes above log_2FC of 1 ie a fold change of at least 2

Table S2. List of European Nucleotide Archive database accession numbers for all nucleotide sequence data used in this study.

			WGS		RNAseq	
**Culture**	**Time Point**	**Replicate**	**Sequencing name**	**Sample accession**	**Sequencing name**	**Run accession (lane1/lane2)**
**C1**	**D0**	1	EM1D0	ERR564134	-	-
		2	EM1D0	ERR303228		
	**D3**	1	EM1D3_COL	ERR564140	-	-
		2	EM1D3	ERR303231		
	**D5**	1	EM1D5_COL	ERR564158	-	-
		2	EM1D5	ERR303233		
	**D10**	1	EM1D10_CAAB	ERR564176	-	-
**C2**	**D0**	1	E-M2D0	ERR564135	EM21D0	ERR576157/ERR576173
		2	EM2D0	ERR303237	EM22D0	ERR576125/ERR576141
	**D3**	1	E-M2D3_COL	ERR564141	EM21D3col	ERR576159/ERR576175
		2	EM2D3	ERR303238	EM22D3col	ERR576127/ERR576143
	**D5**	1	E-M2D5_COL	ERR564159	EM21D5col	ERR576161/ERR576177
		2	EM2D5	ERR303239	EM22D5col	ERR576129/ERR576145
	**D8**	1	D8_EM2_1	ERR777762	-	-
		2	D8_EM2_2	ERR777763		
**C2 Single SNP mutant**	**D5**	-	SNP_col9_LB	ERR757678		

Table S3. List of primers for QPCR and Sanger sequencing used in this study.

Location	Primer name	Sequence 5'->3'	Reference
**QPCR primers**			
BN373_r00191	Kp_16SQ_F	GTTACCCGCAGAAGAAGCAC	70
	Kp_16SQ_R	CTACGCATTTCACCGCTACA	70
BN373_10601	lpxL-2_QF	TTTTCTGACCCTCGAAATGG	70
	lpxL-2_QR	GGTCTTGTTAGAGCGCAAGC	70
BN373_15721	OmpFF	AAAACGGCAACAAACTGGAC	70
	OmpFR	AGACGGGTTTTTGTGGTCTG	70
BN373_06861	lpxA_QF	GGCAACGATAACCTGCTGAT	70
	lpxA_QR	CCATAACGTGTGAGCCAATG	70
BN373_09691	lpxH_QF	CGATGACCAAGGCTATCTGG	70
	lpxH_QR	GTCAACTACCGCCTGTGGAT	70
BN373_45461	OmpAF	AAACATGACCGCGGCAAAGG	This study
	OmpAR	CCCCGGTTCCCTGCATTTTG	This study
BN373_47461	gyrB-F	TTCCTGAACTCCGGCGTCTC	This study
	gyrB-R	CGCTGCGGAATGTTGTTGGT	This study
**PCR primers**			
crrB	Kp_crrB_1_Fw	GTGAATTAACACGCGCATTA	This study
	Kp_crrB_6_Rv	AGTTACAGACGCTGGATGC	This study
